# Supplementary material for: Toroidal diamond anvil cell for detailed measurements under extreme static pressures
Source: Nat Commun. 2018 Jul 25;9:2913. doi: 10.1038/s41467-018-05294-2 (PMC6060175; doi:10.1038/s41467-018-05294-2)
Supplement: Supplementary file 1 — Supplementary Information [file 41467_2018_5294_MOESM1_ESM.pdf]

SUPPLEMENTARY INFORMATION

Toroidal diamond anvil cell for detailed measurements under extreme static pressures

Agnès Dewaele *et al.*

Contents:

Supplementary Figures 1-7
Supplementary Tables 1-7
Supplementary Notes 1-3
Supplementary References

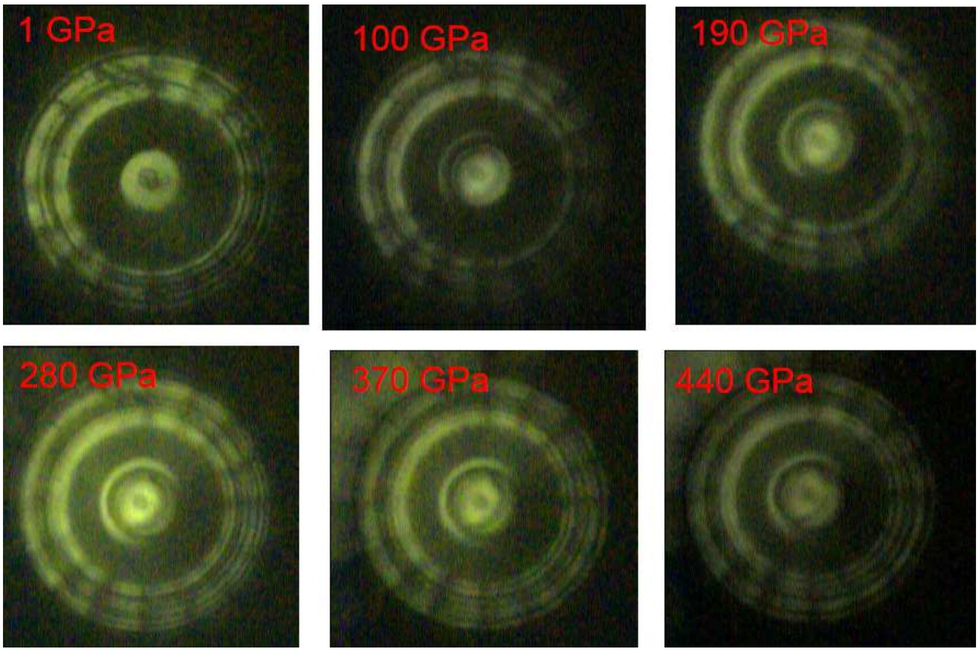

SUPPLEMENTARY FIGURE 1. **Photographs of the sample chamber taken during run 2.** The gold sample (in KCl) is illuminated in reflection.

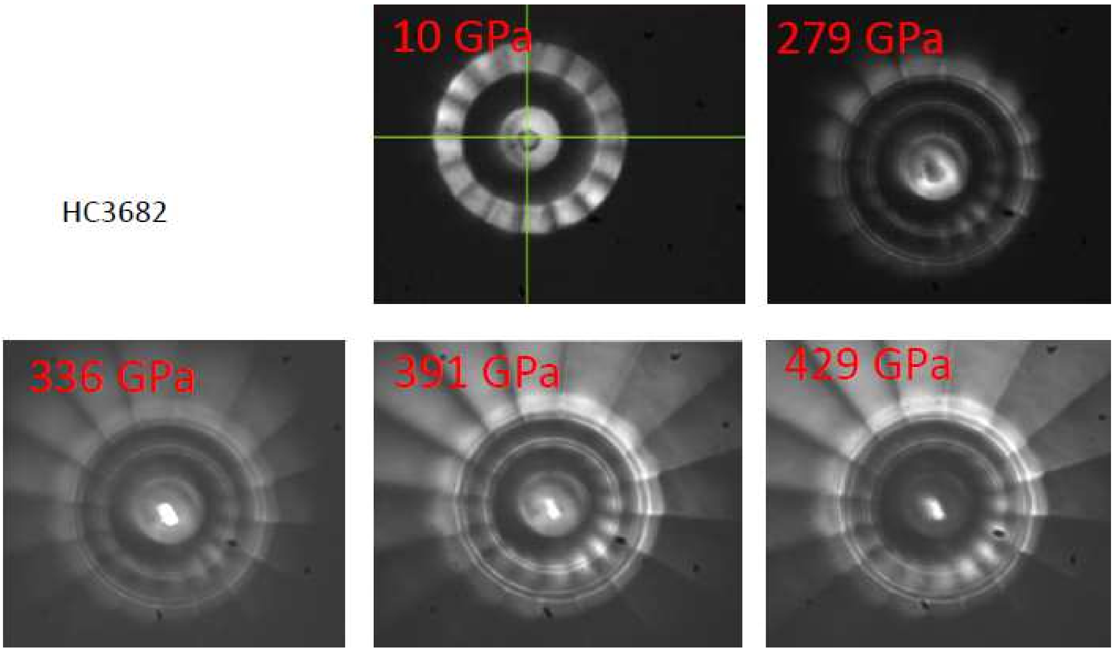

SUPPLEMENTARY FIGURE 2. **Photographs of the sample chamber taken during run 5.** The argon sample is illuminated in reflection and transmission.

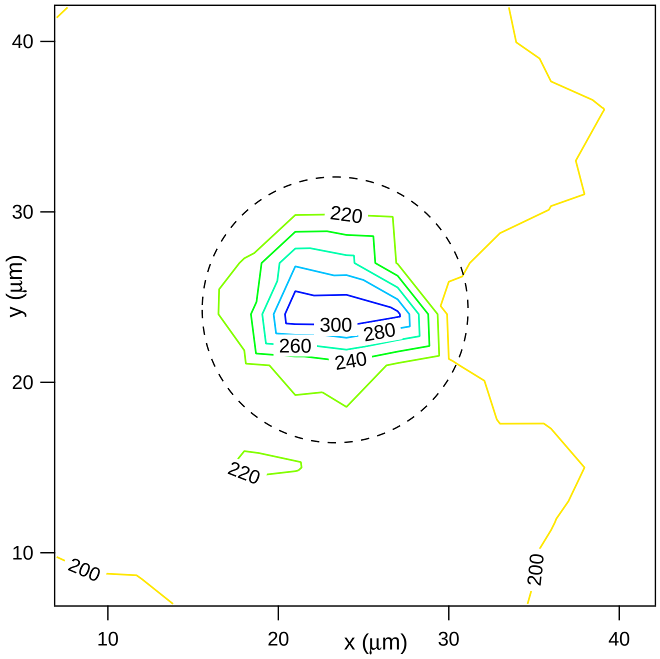

SUPPLEMENTARY FIGURE 3. **Distribution of pressure at the maximum pressure reached in a trial run.** The toroidal pit depth was 5 μm. The pressure reached only 309 GPa in this run. It is estimated using rhenium gasket XRD signal and equation of state [1]. The dashed circle corresponds to the limit of the diamond's central flat. The pressure gradients are high on the central flat (30 GPa/μm).

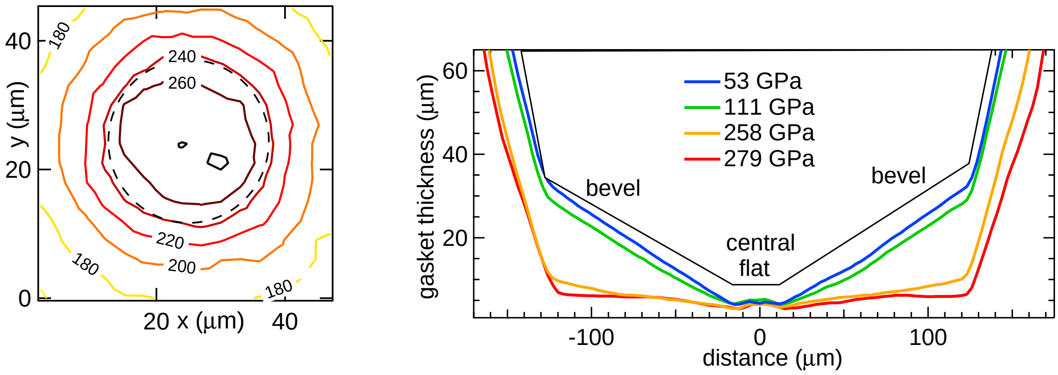

(a) (b)

SUPPLEMENTARY FIGURE 4. (a) **Pressure distribution in a conventional diamond anvil cell.** It is measured every 3μm along a 13×13 points grid, using XRD signal of rhenium gasket with the equation of state from Ref. [1]. The maximum pressure is 279 GPa, and the anvils broke just after this measurement. The dashed circle indicates the limits of the central flat (28 μm diameter). (b) **Strain of the anvils measured at several pressure steps in the same run.** The thickness of the rhenium gasket measured using monochromatic X-ray (λ=0.3738 Å) absorption profiles. The red curve corresponds the data collected just before the anvils breakdown.

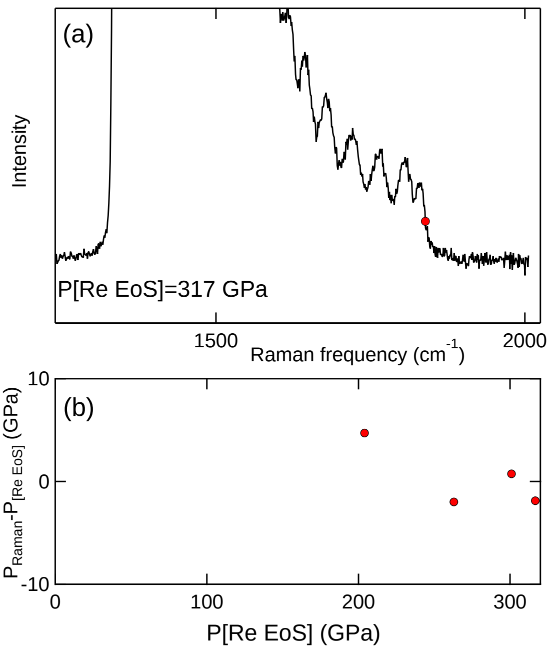

SUPPLEMENTARY FIGURE 5. **(a) Raman spectra collected at the center of the diamond anvil during run 3.** The high frequency edge (red point) ν_C is used to measure the pressure. **(b) Difference between P_{Raman} and the pressure of rhenium gasket P_{Re} in runs 3 and 5.** P_{Raman} is calculated using the calibration valid above 200 GPa in Ref. [2].

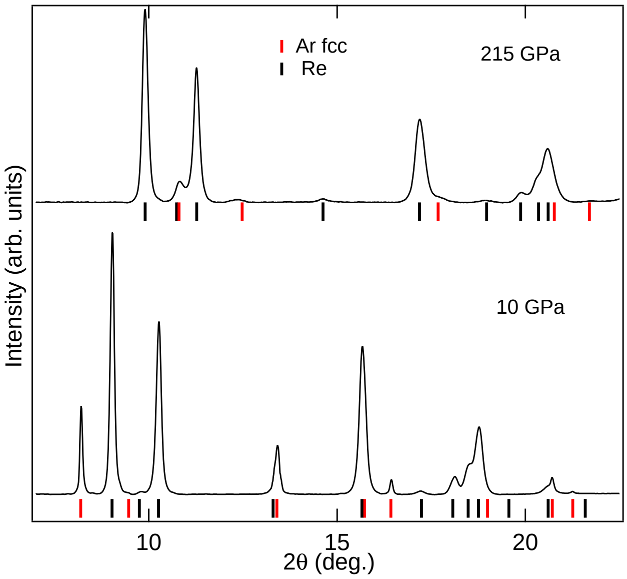

SUPPLEMENTARY FIGURE 6. **XRD spectra collected at the center of the pressure chamber for run 5.** All XRD peaks can be attributed to fcc argon or rhenium diffraction signal, as indicated by tick marks.

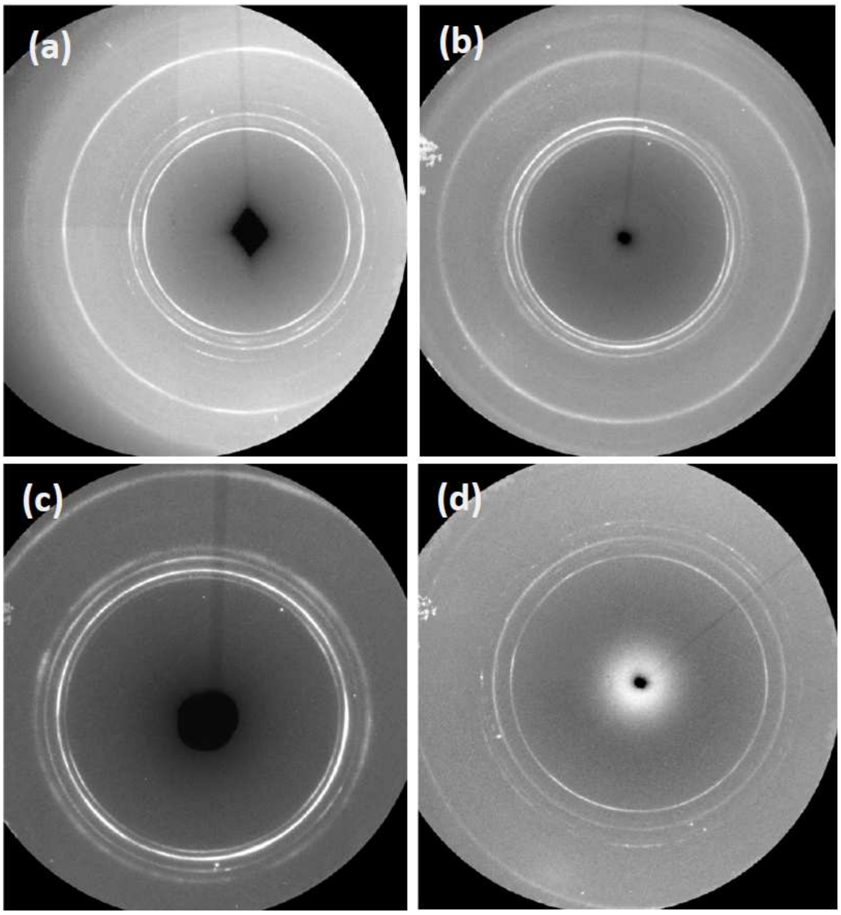

SUPPLEMENTARY FIGURE 7. **X-ray diffraction spectra collected at the center of the toroidal-DAC.** (a) run 1 (rhenium+gold signal), (b) run 2 (rhenium+gold signal), (c) run 3 (rhenium+aluminum+gold signal), (d) run 4 (rhenium+aluminum signal). The detector is a MAR-CCD, and the X-ray wavelength is 0.3738 Å. The exposure time varies between 20 and 40 s.

SUPPLEMENTARY TABLE 1. Raman high frequency edges ν_C measured during runs 3 and 5, for various membrane pressures P_{m}. P_{Raman} are calculated using ν_C and the calibrations from Ref. [2] (BP and HP are the calibrations valid below and above 200 GPa, respectively), and P_{Re} obtained from the rhenium XRD lines and the equation of state in Ref. [1]. For run 5, the anvil Raman signal has been measured in the laboratory four days after the XRD experiment and after a small membrane pressure decrease, so the pressure might have decreased by up to ∼10 GPa between XRD and Raman measurements.

Run	P_{m} (bars)	ν_C (cm^{-1})	P_{Raman} (BP) (GPa)	P_{Raman} (BP) (GPa)	P_{Re} (GPa)
3	32	1701.5	209	205	208
3	37	1771.0	261	261	261
3	42	1821.8	302	311	301
3	44	1837.6	315	327	317
5	170	1918.0	385	425	429

SUPPLEMENTARY TABLE 2. **Equations of state (EoS) parameters obtained by a fit of $P-V$ data for gold, aluminum and argon.** The $P-V$ points plotted in figs. 3, 5 and 6 have been fitted with two equations of state formulations: Rydberg-Vinet (RV) [3] and Holzapfel "H02" [4]. The resulting parameters are listed here, compared with selected literature parameters. The parameters printed in bold have been fixed during the fit. The error bars indicate 95% confidence level of the fit. RV: Rydberg-Vinet [3]; H02: Holzapfel H02 EoS [4]; for argon, these equations of state are evaluated at 0 K (see above). The parameters obtained in this study compare very well with the parameters measured under quasi-hydrostatic compression at lower pressure (Refs. 5–8).

Phase	V_0 (Å^3/at)	K_0 (GPa)	K_0'	EoS formulation	study
Au	**16.962**	**167**	5.94±0.014	RV	this study (0-603 GPa)
Au	**16.962**	**167**	5.74±0.011	H02	this study (0-603 GPa)
Au	**16.962**	**167**	5.9	RV	Ref. 5 (0-131 GPa)
fcc+hcp-Al	16.103±0.2	**73**	4.72±0.18	RV	this study (0-368 GPa)
fcc+hcp-Al	16.207±0.2	**73**	4.41±0.15	H02	this study (0-368 GPa)
fcc-Al	**16.573**	**73**	4.54 ±0.02	RV	Ref. 6 (0-165 GPa)
fcc-Ar	**37.45**	**2.65**	7.562±0.02	RV (0 K) + Debye	this study (0-248 GPa)
fcc-Ar	**37.45**	**2.65**	7.119±0.02	H02 (0 K) + Debye	this study (0-248 GPa)
fcc-Ar	**37.45**	**2.65**	7.587±0.05	RV (0 K) + Debye	Refs. 7 and 8 (0-86 GPa)

SUPPLEMENTARY TABLE 3. **X-ray diffraction lines for aluminum at selected pressures (run 4)** Measured reflections for aluminum in run 4 corresponding to XRD spectra plotted in Fig. 5. $\Delta d = d_{\mathrm{obs}} - d_{\mathrm{calc}}$, d_{obs} and d_{calc} being the interreticular distances, respectively, measured by individual peak fitting and calculated with lattice parameters listed in the table.

Conditions	hkl	d_{obs} (Å)	$\Delta d/d_{\mathrm{obs}}$ ($\times 10^4$)
fcc-Al, a=3.2506 Å	111	1.877	1.7
P_{Re}=184 GPa	200	1.625	-1.7
fcc-Al, a=3.1827 Å	111	1.837	-2.7
P_{Re}=231 GPa	200	1.592	2.7
hcp-Al, a=2.2504 Å, c=3.672 Å	100	1.949	0.5
	002	1.836	0.0
	101	1.721	-2.9
	102	1.337	4.4
hcp-Al, a=2.200 Å, c=3.5674 Å	002	1.784	1.7
P_{Re}=301 GPa	101	1.681	2.4
	102	1.301	-8.4
hcp-Al, a=2.1625 Å, c=3.485 Å	002	1.742	-2.9
P_{Re}=301 GPa	101	1.649	-2.4
	102	1.277	10.1
bcc-Al	110	1.704	-

SUPPLEMENTARY TABLE 4. **Au EoS data.** Lattice parameters measured in runs 1 and 2. $P_{\rm m}$ (in bars) is the membrane pressure. The rhenium lattice parameters have been determined by a fit of XRD peaks positions with c/a ratio fixed to the average value obtained by a least-square refinement af all data (c/a=1.604 and 1.609 in runs 1 and 2). For fcc Au, (111) and (200) diffraction peaks have been measured. $P_{\rm Re}$ (in GPa) is the pressure measured using Re lattice parameters and EoS [1]. The data are listed in the order they have been taken. Atomic volumes are $V = a_{111\ \rm Au}^3/4$ for Au and $V = \sqrt{3} \times a_{\rm Re}^2 \times c_{\rm Re}/4$ for Re.

Run	$P_{\rm m}$	$a_{\rm Re}$ (Å)	$c_{\rm Re}$ (Å)	$P_{\rm Re}$	$a_{111\ \rm Au}$ (Å)	$a_{200\ \rm Au}$ (Å)	Run	$P_{\rm m}$	$a_{\rm Re}$ (Å)	$c_{\rm Re}$ (Å)	$P_{\rm Re}$	$a_{111\ \rm Au}$ (Å)	$a_{200\ \rm Au}$ (Å)
1	15	2.7427	4.3993	10.0	4.0093	4.0099	2	33	2.7423	4.4124	9.30	4.0398	4.0378
	25	2.7313	4.3810	16.0	3.9775	3.9766		36	2.7404	4.4094	10.2	4.0261	4.0256
	35	2.7131	4.3518	24.0	3.9269	3.9261		39	2.7352	4.4010	12.5	4.0097	4.0098
	45	2.6890	4.3131	37.0	3.8650	3.8780		41	2.7293	4.3915	15.2	3.9891	3.9921
	50	2.4852	3.9863	232	3.5314	3.5249		43	2.7276	4.3888	15.9	3.9825	3.9884
	51	2.4773	3.9735	243	3.5251	3.5236		45	2.7248	4.3842	17.3	3.9716	3.9800
	53	2.4595	3.9450	271	3.5001	3.4993		47	2.7209	4.3779	19.1	3.9603	3.9709
	53	2.4541	3.9363	280	3.4920	3.4875		49	2.7176	4.3725	20.8	3.9506	3.9613
	55	2.4503	3.9303	286	3.4868	3.4817		51	2.7131	4.3654	22.9	3.9389	3.9509
	57	2.4338	3.9039	315	3.4663	3.4615		53	2.7108	4.3616	24.1	3.9270	3.9398
	57	2.4346	3.9052	314	3.4651	3.4619		55	2.7022	4.3478	28.6	3.9086	3.9212
	60	2.4222	3.8852	337	3.4479	3.4468		57	2.6727	4.3005	45.3	3.8339	3.8476
	63	2.4110	3.8673	358	3.4320	3.4308		57	2.6495	4.2630	60.4	3.7805	3.7994
	66	2.4025	3.8536	376	3.4218	3.4217		57	2.6457	4.2569	63.0	3.7745	3.7954
	69	2.3946	3.8410	392	3.4112	3.4108		58	2.6346	4.2391	70.9	3.7545	3.7776
	72	2.3883	3.8308	406	3.4021	3.4012		59	2.5834	4.1567	113	3.6698	3.6954
	75	2.3874	3.8294	408	3.4002	3.4032		60	2.4981	4.0194	210	3.5511	3.5706
	78	2.3785	3.8152	428	3.3900	3.3890		61	2.4976	4.0187	211	3.5520	3.5785
	82	2.3733	3.8067	440	3.3830	3.3869		62	2.4975	4.0185	211	3.5520	3.5770
	86	2.3678	3.7979	453	3.3765	3.3817		63	2.4927	4.0107	217	3.5450	3.5685
	90	2.3651	3.7935	459	3.3693	3.3744		64	2.4836	3.9961	230	3.5349	3.5600
	94	2.3585	3.7830	475	3.3623	3.3698		65	2.4671	3.9696	255	3.5148	3.5423
	98	2.3547	3.7769	485	3.3569	3.3634		66	2.4509	3.9435	281	3.4947	3.5242
	103	2.3517	3.7721	493	3.3527	3.3544		67	2.4419	3.9291	296	3.4827	3.5131
	108	2.3485	3.7669	501	3.3487	3.3566		67	2.4430	3.9307	294	3.4823	3.5163
	113	2.3450	3.7613	510	3.3443	3.3469		72	2.4229	3.8984	331	3.4555	3.4919
	118	2.3424	3.7573	517	3.3396	3.3476		75	2.4138	3.8838	348	3.4439	3.4894
	123	2.3382	3.7504	528	3.3349	3.3367		80	2.4015	3.8640	373	3.4270	3.4784
	129	2.3333	3.7426	541	3.3298	3.3245		85	2.3923	3.8492	392	3.4155	3.4710
	135	2.3319	3.7404	545	3.3261	3.3248		90	2.3842	3.8362	409	3.4042	3.4574
	141	2.3281	3.7343	556	3.3225	3.3198		100	2.3693	3.8122	443	3.3849	3.4422
	147	2.3254	3.7299	563	3.3190	3.3180							
	154	2.3221	3.7246	573	3.3146	3.3127							
	161	2.3189	3.7196	582	3.3116	3.3120							
	170	2.3154	3.7138	593	3.3068	3.3034							
	180	2.3117	3.7080	603	3.3019	3.3005							

SUPPLEMENTARY TABLE 5. **Au EoS data.** Lattice parameters measured in run 3. P_{m} (in bars) is the membrane pressure. a_{Re} and c_{Re} have been determined using a fixed c/a ratio (1.613 below 250 GPa, 1.606 above). a_{111} is the cubic lattice parameter measured using the (111) diffration peak. P_{Re} (in GPa) is the pressure measured using Re lattice parameters and EoS [1]. The data are listed in the order they have been taken. Atomic volumes are $V = a_{111}^3/4$ for fcc lattices and $V = \sqrt{3} \times a^2 \times c/4$ for hcp lattices.

Run	P_{m}	a_{Re} (Å)	c_{Re} (Å)	P_{Re}	$a_{111\ \mathrm{Au}}$ (Å)	$a_{111\ \mathrm{Al\ fcc}}$ (Å)	$a_{\mathrm{Al\ hcp}}$ (Å)	$c_{\mathrm{Al\ hcp}}$ (Å)
3	15	2.7477	4.4320	6.1	4.0531	3.9893		
	20	2.7369	4.4147	10.7	4.0059	3.9045		
	24	2.7257	4.3966	15.8	3.9755	3.8345		
	28	2.7167	4.3820	20.1	3.9513	3.7840		
	32	2.5313	4.0830	165	3.6100	3.3006		
	32	2.5024	4.0267	204	3.5657	3.2444		
	34	2.4951	4.0246	211	3.5592	3.232		
	35	2.4891	4.0149	219	3.5503	3.219		
	36	2.4785	3.9980	235	3.5329	3.200	2.265	3.671
	37	2.4640	3.9550	263	3.5100		2.246	3.621
	38	2.4590	3.9484	270	3.5041		2.240	3.613
	39	2.4560	3.9435	275	3.4943		2.234	3.605
	40	2.4490	3.9322	287	3.4881		2.227	3.594
	41	2.4450	3.9260	294	3.4824		2.224	3.584
	42	2.4410	3.9190	301	3.4772		2.221	3.576
	43	2.4360	3.9120	309	3.4730		2.214	3.565
	44	2.4320	3.9054	317	3.4631		2.209	3.558

SUPPLEMENTARY TABLE 6. **Al EoS data.** Lattice parameters measured in run 4. P_{m} (in bars) is the membrane pressure. a_{Re} and c_{Re} have been determined using a fixed c/a ratio (1.61 below 250 GPa, 1.602 above). a_{111} is the cubic lattice parameter measured using the (111) diffration peak. P_{Re} (in GPa) is the pressure measured using Re lattice parameters and EoS [1]. The data are listed in the order they have been taken. Atomic volumes are $V = a_{111}^3/4$ for fcc lattices, $V = \sqrt{3} \times a^2 \times c/4$ for hcp lattices and $V = a^3/2$ for the bcc lattice.

Run	P_{m}	a_{Re} (Å)	c_{Re} (Å)	P_{Re}	$a_{111\ \mathrm{Al\ fcc}}$ (Å)	$a_{\mathrm{Al\ hcp}}$ (Å)	$c_{\mathrm{Al\ hcp}}$ (Å)	$a_{\mathrm{bcc\ Al}}$ (Å)
4	28	2.748	4.432	6.12	4.011			
	32	2.743	4.424	8.10	3.988			
	33	2.743	4.424	8.28	3.977			
	35	2.742	4.423	8.51	3.954			
	37	2.741	4.421	8.97	3.933			
	38	2.734	4.410	12.0	3.912			
	40	2.730	4.403	13.9	3.894			
	42	2.724	4.394	16.6	3.869			
	44	2.723	4.392	17.1	3.850			
	46	2.720	4.390	18.2	3.828			
	48	2.716	4.382	20.3	3.803			
	50	2.710	4.372	23.3	3.780			
	52	2.707	4.367	24.8	3.769			
	54	2.710	4.361	24.5	3.752			
	56	2.696	4.341	31.5	3.690			
	57	2.660	4.284	52.8	3.582			
	57.5	2.659	4.281	53.7	3.572			
	58	2.651	4.289	56.1	3.550			
	58	2.636	4.244	69.5	3.513			
	58.5	2.619	4.217	82.3	3.459			
	59	2.609	4.201	90.3	3.430			
	59	2.595	4.180	102	3.393			
	59.5	2.585	4.163	111	3.379			
	61	2.556	4.116	140	3.312			
	62	2.537	4.085	161	3.275			
	63	2.516	4.059	184	3.252			
	64	2.505	4.040	198	3.235	2.285	3.682	
	65	2.496	4.027	210	3.221	2.277	3.670	
	65	2.489	4.014	220	3.204	2.266	3.667	
	66	2.487	4.011	223	3.198	2.264	3.664	
	67	2.485	4.008	225	3.192	2.259	3.656	
	68	2.481	4.001	231	3.181	2.252	3.644	
	69	2.481	3.973	240	3.170	2.246	3.642	
	70	2.475	3.964	249	3.162	2.241	3.634	
	72	2.468	3.953	259		2.230	3.617	
	74	2.459	3.938	274		2.220	3.609	
	76	2.454	3.929	283		2.212	3.596	
	78	2.448	3.920	293		2.205	3.583	
	80	2.443	3.912	301		2.200	3.568	
	83	2.437	3.903	312		2.193	3.556	
	83	2.433	3.897	319		2.188	3.549	
	86	2.430	3.892	324		2.186	3.545	
	89	2.426	3.886	332		2.180	3.535	
	92	2.424	3.881	336		2.174	3.527	
	95	2.417	3.871	349		2.168	3.516	2.423
	98	2.414	3.866	355		2.164	3.510	2.417
	100	2.410	3.859	363		2.161	3.503	2.413
	103	2.407	3.856	368		2.159	3.494	2.411

SUPPLEMENTARY TABLE 7. **Ar EoS data.** Lattice parameters measured in run 5. P_{m} is the membrane pressure. a_{Re} and c_{Re} have been determined using a fixed c/a ratio (1.613 below 250 GPa, 1.606 above). $a_{111;Ar}$ is the cubic lattice parameter of argon measured using the (111) diffration peak. P_{Re} is the pressure measured using Re lattice parameters and EoS [1]. The data are listed in the order they have been taken. Atomic volumes are $V = a_{111\,\mathrm{Ar}}^3/4$ for argon and $V = \sqrt{3} \times a_{\mathrm{Re}}^2 \times c_{\mathrm{Re}}/4$ for Re.

Run	P_{m} (bars)	a_{Re} (Å)	c_{Re} (Å)	P_{Re} (GPa)	$a_{111\,\mathrm{Ar}}$ (Å)
5	42	2.744	4.398	10.3	4.531
	47	2.739	4.393	12.0	4.489
	52	2.735	4.386	14.0	4.389
	57	2.730	4.377	16.5	4.296
	62	2.723	4.376	18.6	4.224
	67	2.656	4.259	58.1	3.857
	67	2.612	4.180	92.2	3.712
	70	2.566	4.115	133	3.600
	72	2.535	4.065	167	3.520
	74	2.519	4.021	191	3.479
	76	2.501	3.993	215	3.439
	78	2.495	3.983	223	3.421
	80	2.478	3.957	248	3.384
	83	2.473	3.948	255	
	86	2.464	3.933	270	
	89	2.459	3.925	279	
	94	2.449	3.909	296	
	99	2.438	3.900	312	
	104	2.430	3.889	325	
	109	2.423	3.885	336	
	115	2.416	3.874	349	
	123	2.408	3.856	367	
	131	2.402	3.851	377	
	140	2.396	3.841	391	
	150	2.389	3.83	405	
	160	2.384	3.822	416	
	170	2.378	3.813	429	

SUPPLEMENTARY NOTE 1: RAMAN SIGNAL OF THE DIAMOND ANVILS (RUNS 3 AND 5)

The pressure based on diamond anvil fluorescence signal measured at several pressure steps in run 3. The Raman signal of the diamond anvil in contact with the sample has been excited with a 30 mW laser with 647 nm wavelength, collected by a $\times 50$ microscope objective and dispersed with a Shamrock 1200 grating spectrometer, equipped with an Andor iDus 401 camera; one spectrum is presented in Supplementary Figure 5a. The calibration is from Ref. [2] (calibration valid below 200 GPa). The pressure is compared with the pressure measured with rhenium EoS [1] in Supplementary Figure 5b. We find that the calibration expected to be valid above 200 GPa in Ref. [2] slightly overestimates pressure (by up to 12 GPa) in run 3 but agrees with rhenium pressure in run 5 (within error bars, see Supplementary Table 1). The current measurements thus suggest that rhenium [1] and Raman HP [2] pressure gauges agree within 12 GPa up to 430 GPa.

SUPPLEMENTARY NOTE 2: EVALUATION OF NON-HYDROSTATIC STRESS (RUNS 1 AND 2)

We provide below the details of the analysis carried out to estimate $\sigma_3 - \sigma_1$, where σ_3 and σ_1 are the stress components in the axial (vertical) and radial directions in the sample, respectively.

Analysis of the positions of gold X-ray diffraction lines

The non-hydrostatic stress is evaluated using the method developed by Singh [9] and commonly used in the high pressure literature [5, 10]. For cubic crystals, it is based on the comparison between the lattice parameters calculated from the diffraction angle for different diffraction lines. If the crystal remains cubic, all diffraction lines yield identical lattice parameters. If it is strained, they differ by a factor proportional to $\sigma_3 - \sigma_1$ and to the elastic anisotropy of the crystal S:

$$S = (1/C' - 1/C)/2, \tag{1}$$

where $C = C_{44}$ and $C' = (C_{11} - C_{12})/2$ are the two shear constants of the cubic crystal. S was estimated following the same method as in Ref. 5 based on low compression measurements of the elastic moduli of gold [11].

In runs 1 and 2, we could follow two gold diffraction lines: (111) and (200). For these lines, Singh's analysis reduces to (with an error of less than 4 %):

$$\sigma_3 - \sigma_1 = \frac{3(a_{200} - a_{111})}{\alpha a_{111} S}, \tag{2}$$

where α is a factor between 0.5 and 1. We plotted in Fig. 3 the results obtained for α=1, which corresponds to an iso-stress assumption in the powder and provides minimum values for $\sigma_3 - \sigma_1$.

Analysis of the X-ray diffraction lines width

We followed the method proposed in Ref. [12]. The micro-stress σ is the difference in stress between the crystallites that constitute the powder. This stress difference is upper bounded by the yield strength of the material. σ is expressed as a function of X-ray diffraction peaks angle (2θ is the diffraction angle) and Full Width at Half Maximum, FWHM, through:

$$(FWHM \cos\theta)^2 = (2\sigma)^2 \frac{\sin^2\theta}{E_{\rm hkl}^2} + (FWHM(\sigma = 0)\cos\theta)^2, \tag{3}$$

$E_{\rm hkl}$ being the single crystal Young modulus along the direction hkl at the relevant pressure. For a cubic material [13]:

$$E_{\rm hkl} = \left(\frac{C' + 3K}{9KC'} - \frac{C - C'}{CC'} \Gamma_{\rm hkl} \right)^{-1}, \tag{4}$$

where K is the bulk modulus of the crystal, C and C' are the shear constants of the cubic crystal (defined above), calculated using literature data [5, 11], and $\Gamma_{\rm hkl}$ is defined by:

$$\Gamma_{\rm hkl} = (h^2k^2 + h^2l^2 + k^2l^2)/(h^2 + k^2 + l^2)^2. \tag{5}$$

The measurement of $FWHM$ for two diffraction lines of gold, (111) and (200), allows to evaluate σ as plotted in Fig. 3.

SUPPLEMENTARY NOTE 3: EQUATION OF STATE OF ARGON FROM LITERATURE DATA

Following Finger et al. [7], the pressure in argon is expressed as a sum of a static pressure $P_0(V)$ (expressed with a Rydberg-Vinet equation of state [3]) at 0 K and a thermal pressure $P_{th}(V,T)$ estimated with the Mie-Grüneisen Debye formalism: $P(V,T) = P_0(V) + P_{th}(V,T)$. $x = V/V_0$, V_0 being the equilibrium volume of argon ar ambient pressure and 0 K.

$$P_0(V) = 3K_0(1-x)x^{-2}\exp\left(\frac{3}{2}(K_0'-1)(1-x)\right), \tag{6}$$

and

$$P_{th}(V,T) = \frac{3\gamma RT}{V}\left[D\left(\frac{\theta_D}{T}\right)\right]. \tag{7}$$

(the zero point pressure is neglected). In Eq. 6, the parameters K_0 and K_0' are the bulk modulus and its pressure derivative at equilibrium volume and 0 K. In Eq. 7, D is the Debye function and the Grüneisen parameter γ is assumed to behave as [7]:

$$\gamma(V) = 2.20 \times x + 0.5 \tag{8}$$

and the Debye temperature as:

$$\theta_D(V) = 93.3\sqrt{(}1/x) \times \exp(2.20(1-x)). \tag{9}$$

We have fixed V_0 and K_0 to the values given in Refs. 14 and 15: $V_0 = 37.45$ Å^3/at and $K_0 = 2.65$ GPa. The parameter K_0' has been obtained by a fit of the 298 K data reported in Refs. 7 and 8, with the ruby pressure modified according to the correction proposed in Ref. 16: $K_0' = 7.587$.

SUPPLEMENTARY REFERENCES

[1] Anzellini, S., Dewaele, A., Occelli, F., Loubeyre, P. & Mezouar, M. Equation of state of rhenium and application for ultra high pressure calibration. *J. Appl. Phys.* **115**, 043511 (2014).

[2] Akahama, Y. & Kawamura, H. Pressure calibration of diamond anvil Raman gauge to 410 GPa. In Takemura, K (ed.) *International conference on high pressure science and technology, joint AIRAPT-22 and HPCJ-50*, vol. 215 of *Journal of Physics Conference Series*, 012195 (2010). Joint AIRAPT-22 and HPCJ-50 Conference/International Conference on High Pressure Science and Technology, Tokyo, JAPAN, JUL 26-31, 2009.

[3] Vinet, P., Rose, J. H., Ferrante, J., & Smith, J. R. Universal features of the equation of state of solids. *J. Phys. Condens. Matter* **1**, 1941–1963 (1989).

[4] Holzapfel, W. Equations of State for regular solids. *High Press. Res.* **22**, 209–216 (2002). 39th European High Pressure Research Group Meeting on Advanves on High Pressure Research (EHPRHG'39), SANTANDER, SPAIN, SEP 16-19, 2001.

[5] Takemura, K. & Dewaele, A. Isothermal equation of state for gold with a He-pressure medium. *Phys. Rev. B* **78**, 104119–104131 (2008).

[6] Dewaele, A., Loubeyre, P. & Mezouar, M. Equations of state of six metals above 94 GPa. *Phys. Rev. B* **70**, 094112–094119 (2004).

[7] Finger, L., Hazen, R., Zou, G., Mao, H., H. & Bell, P. Structure and compression of crystalline argon and neon at high-pressure and room-temperature. *Appl. Phys. Lett.* **39**, 892–894 (1981).

[8] Ross, M., M, Mao, H., Bell, P. & Xu, J. A. The equation of state of dense argon - a comparison of shock and static studies. *J. Chem. Phys.* **85**, 1028–1033 (1986).

[9] Singh, A. & Kenichi, T. Measurement and analysis of nonhydrostatic lattice strain component in niobium to 145 GPa under various fluid pressure-transmitting media. *J. Appl. Phys.* **90**, 3269–3275 (2001).

[10] Dewaele, A. & Loubeyre, P. Pressurizing conditions in helium-pressure-transmitting medium. *High Press. Res.* **27**, 419–429 (2007).

[11] Hiki, Y. & Granato, A. V. Anharmonicity in noble metals; higher order elastic constants. *Phys. Rev.* **144**, 411–419 (1966).

[12] Singh, A. K., Liermann, H. P. & Saxena, S. K. Strength of magnesium oxide under high pressure: Evidence for the grain-size dependence. *Sol. State Comm.* **132**, 795 (2004).

[13] Nagao, S., Nordlund, K. & Nowak, R. Anisotropic elasticity of IVB transition-metal mononitrides determined by ab initio calculations. *Phys. Rev. B* **73**, 144113 (2006).

[14] Peterson, O., Batchelder, D. & Simmons, R. Measurements of X-ray lattice constant thermal expansivity and isothermal compressibility of argon crystals. *Phys. Rev.* **150**, 703 (1966).

[15] Jelinek, G. Properties of crystalline argon, krypton, and xenon based upon Born-Huang method of homogeneous deformations. III. The low-temperature limit. *Phys. Rev. B* **5**, 3210 (1972).

[16] Dewaele, A., Torrent, M., Loubeyre, P. & Mezouar, M. Compression curves of transition metals in the Mbar range: Experiments and projector augmented-wave calculations. *Phys. Rev. B* **78**, 104102–104114 (2008).
